# Sport trainings as a stress prophylactic mean during COVID-19 pandemic

**DOI:** 10.1192/j.eurpsy.2022.1340

**Published:** 2022-09-01

**Authors:** M. Titova, V. Pulkina

**Affiliations:** Lomonosov Moscow State University, Department Of Psychology, Moscow, Russian Federation

**Keywords:** COVID-19, stress, trait anxiety, sport tranings

## Abstract

**Introduction:**

COVID-19 pandemic assumed as an additional stress factor for people due to extraordinary work conditions, unclear expectations of the future, anxiety about the self-health and health of close people (Samanta et al., 2020; Pascale, 2020). Sport training can be considered as a mean of stress consequences prevention during COVID-19 pandemic (Jimеnez-Pavоn et al., 2020). It is known that moderate physical loads are related to strengthening the immune system and reducing the risk of disease, depression, anxiety (Landers, 1996; Schwellnus et al., 2016). Some authors recommend to maintain an active lifestyle in the COVID-19 period (Mattioli et al, 2020).

**Objectives:**

The study was held in 62 professionals from different fields, who work remotely during the self-isolation due to COVID-19 pandemic and aimed to estimate sports trainings opportunities as a means of preventing stress of professionals in various fields of activities during the COVID-19 pandemic.

**Methods:**

The assessment methods included: 1) author’s questionnaire about the attitude towards sports trainings; 2) A.B. Leonova’s “Chronic fatigue” and “Chronic stress”; 3) Ch. Spilberger’s “Trait anxiety”.

**Results:**

The results revealed that the low level of chronic stress (U=82; p=0,015), chronic fatigue (U=82; p=0,015) and trait anxiety (U=79; p=0,011) is more typical for those surveyed who experienced COVID-19 symptoms and engaged in sports trainings with moderate loads than those people with COVID-19 symptoms who did not attend sport trainings.

**Conclusions:**

The results of the study can be used to develop programs to improve the psychological well-being and performance of professionals working under stress due to COVID-19 pandemic.

**Disclosure:**

No significant relationships.

